# Vessel Enlargement in Development and Pathophysiology

**DOI:** 10.3389/fphys.2021.639645

**Published:** 2021-02-25

**Authors:** Laia Gifre-Renom, Elizabeth A. V. Jones

**Affiliations:** ^1^Department of Cardiovascular Sciences, Centre for Molecular and Vascular Biology, KU Leuven, Leuven, Belgium; ^2^Department of Cardiology, CARIM School for Cardiovascular Diseases, Maastricht University, Maastricht, Netherlands

**Keywords:** venogenesis, migration, vascular fusion, mechanotransduction, collateral growth, arteriogenesis, vessel enlargement, arterial venous malformation

## Abstract

From developmental stages until adulthood, the circulatory system remodels in response to changes in blood flow in order to maintain vascular homeostasis. Remodeling processes can be driven by *de novo* formation of vessels or angiogenesis, and by the restructuration of already existing vessels, such as vessel enlargement and regression. Notably, vessel enlargement can occur as fast as in few hours in response to changes in flow and pressure. The high plasticity and responsiveness of blood vessels rely on endothelial cells. Changes within the bloodstream, such as increasing shear stress in a narrowing vessel or lowering blood flow in redundant vessels, are sensed by endothelial cells and activate downstream signaling cascades, promoting behavioral changes in the involved cells. This way, endothelial cells can reorganize themselves to restore normal circulation levels within the vessel. However, the dysregulation of such processes can entail severe pathological circumstances with disturbances affecting diverse organs, such as human hereditary telangiectasias. There are different pathways through which endothelial cells react to promote vessel enlargement and mechanisms may differ depending on whether remodeling occurs in the adult or in developmental models. Understanding the molecular mechanisms involved in the fast-adapting processes governing vessel enlargement can open the door to a new set of therapeutical approaches to be applied in occlusive vascular diseases. Therefore, we have outlined here the latest advances in the study of vessel enlargement in physiology and pathology, with a special insight in the pathways involved in its regulation.

## Introduction

During development, vascular beds often form as honeycombed shaped capillary plexus that then become perfused and remodel to form a hierarchical vessel structure. One of the most visible changes that occur in this process is the enlargement of both arteries and veins. Vessel enlargement can occur remarkably quickly. In the embryo, this occurs within a single day after the onset of blood flow. As such, vessel enlargement has therapeutic potential in occlusive vascular diseases that slower processes, such as angiogenesis, lack. However, so far, most research on therapeutic strategies has been focused on angiogenesis and significantly less advancement has been made to exploit vessel enlargement as a potential therapy in ischemic diseases. In strokes and heart attacks, for example, local collateral vessels immediately dilate to restore blood flow, but angiogenesis is a latter process in the body’s attempt to restore proper perfusion (Schaper and Ito, 1996). Vessel can dilate on the short term, but can also undergo outward remodeling such that vessel diameter changes are permanent (Silver and Vita, 2006). As such, it is primarily vessel enlargement that prevents excessive cell death.

Though occlusive vascular diseases are important pathologies where inducing vessel enlargement could provide therapeutic benefits, when vessels enlarge ectopically, there can be devastating negative consequences. Arterial-venous malformations (AVMs) are a form of anomalous vessel enlargement where enlarged shunts bypass the capillary bed and directly connect arteries and veins. Because the venous network is then exposed to arterial blood pressure, AVMs are prone to hemorrhage, which can be fatal depending on the organ where they occur. For instance, cerebral AVMs account for 50% of childhood strokes (Meyer-Heim and Boltshauser, 2003). Though AVMs are the most serious example of pathological vessel enlargement, these are not the only example of such a process. Varicose veins also represent a form of pathological vessel enlargement (Jacobs et al., 2017) that can cause itching and discomfort, and are one of the most common reported medical conditions in Western countries (Beebe-Dimmer et al., 2005).

Despite the importance of vessel enlargement, we are just beginning to understand how diameter changes occur. It was initially assumed that vessels enlarged by proliferation and while this may be true in some vascular beds, proliferation is a slow process and therefore could not restore blood flow on the timescales needed after stroke or heart attack. Vessel dilation, followed by outward remodeling can, at least partially, mitigate the slow process of cell proliferation. More recently, however, migration and capillary fusion have been proposed as methods by which a vascular network can increase the diameter of vessels. It is important to note, however, that it is unlikely that any of these processes happen in isolation. As such, vascular networks likely use a combination of means to increase vessel diameters. We therefore review here the processes and pathways by which vessels enlarge, and highlight differences and similarities between vascular development, vasculopathy and enlargement in adult vascular beds.

## Mechanisms of Vessel Enlargement

Four mechanisms of vessel enlargement have been identified, though the prevalence or relative importance of each of these mechanisms is still not clear. First, endothelial cell migration can lead to the accumulation of cells in one region, leading to regional enlargement of a vessel. Second, smaller vessels can fuse with each other, thereby rapidly increasing the diameter. Vessels can also increase in diameter because of either local proliferation of endothelial cells (third mechanism), or by local hypertrophy of endothelial cells (fourth). Vessel enlargement occurring in developmental vascular networks refers to the enlargement of capillaries to form arterioles/venules. In the adult, vessel enlargement has mostly only been studied with respect to enlargement of larger vessels (arterioles and venules) and of collateral vessels. Differences in the type of vessel which is enlarging can also account for differences in mechanism highlighted below.

### Migration

**Figure 1:**
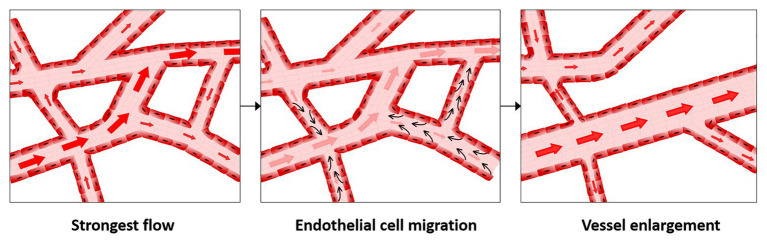
Migration-induced vessel enlargement. Under low shear stress, endothelial cells migrate against flow and stop or reduce migration when exposed to shear stresses at or above their shear stress physiological levels. In this way, endothelial cells accumulate in vessels under higher shear stress, leading to vessel enlargement.

Migration is currently the most well accepted mechanism for vessel enlargement in developmental models. In this mechanism, vessel enlargement occurs by a reshuffling of existing vessels guided by changes in shear stress. Shear stress is the force per unit area exerted by flow, expressed either in N/m^2^ (i.e., Pascals) or in dyne/cm^2^. Shear stress can be thought of as friction against the endothelium. It relates both to the speed of the blood flow and vessel geometry. In the migration paradigm of vessel enlargement, endothelial cells migrate against flow when shear stress levels are decreased compared to physiological normal levels (which are 15 dyn/cm^2^ in humans or 30 dyn/cm^2^ in mouse) and stop migrating in the presence of physiological levels (Franco et al., 2015; Tabibian et al., 2020). Therefore, vessels with the lowest flow rates regress increasing the pool of available endothelial cells. Furthermore, because endothelial cells stop migrating in high shear stress vessels, they accumulate in regions of high shear stress (Figure 1). This idea was first proposed approximately 20 years ago (Hughes and Chang-Ling, 2000) but gained significantly more interest as imaging technology improved, allowing individual endothelial cell tracking (Udan et al., 2013; Franco et al., 2015).

In support of this mechanism, multiple groups have shown that there is very little proliferation or apoptosis during vessel remodeling and vessel enlargement, either in the retina, in zebrafish embryo or in mouse embryo (Hughes and Chang-Ling, 2000; Udan et al., 2013; Franco et al., 2015). Myosin light chain 2a (*MLC2a*)^−/−^ embryos, which have severely reduced flow and do not undergo vessel enlargement, have the same levels of endothelial cell proliferation and apoptosis as control wild-type embryos (Udan et al., 2013). Furthermore, time-lapse microscopy of developing embryos has shown that there is a stunning amount of endothelial cell migration occurring during vascular remodeling (Sato et al., 2010; Cui et al., 2013). Using a quail embryo that has Yellow Fluorescent Protein (YFP)-labeled endothelial cells, Cui et al. (2013) imaged vitelline artery formation (see especially movie S4). Endothelial cells can be observed migrating against flow, towards the embryo proper, as a mass collective movement. Similarly, using time-lapse microscopy of whole mouse embryos between embryonic day (E) E8.5 and E9.5, Udan et al. (2013) showed that endothelial cells leave low flow capillaries towards enlarging vessels, whereas in the *MLC2a*^−/−^ mutant embryos, endothelial cells lose their ability to undergo directional migration.

Our group has recently used the migration-induced mechanism of vessel enlargement to develop computational models of remodeling and vessel enlargement (Tabibian et al., 2020). *In vitro*, we found that there is indeed a bell-shaped pattern of migration with respect to shear stress. Endothelial cells do not migrate in static or at extremely low shear stress levels. Endothelial cells exposed to shear stress levels between 0.5 and 5 dyn/cm^2^ show the highest level of migration, with a peak at 1 dyn/cm^2^. Higher shear stress levels return migration rates to static levels (Tabibian et al., 2020). This information was then used to build the computational model where shear-stress levels defined the speed of migration, but the direction of migration was influenced both by the direction of the flow and a requirement for collective cell movement. This alone gave modest predictive ability, which was improved by the addition of growth of avascular regions and, more surprisingly, by the addition of endothelial cell elongation in the direction of flow. Although the role of cell elongation in remodeling is currently unexplored, studies on mice where endothelial cells cannot elongate did not report vessel enlargement defects (Baeyens et al., 2014; Corti et al., 2019). Those results, however, also found that shear stress magnitude is sensed apart from shear stress direction (Baeyens et al., 2014), consistent with our computational results.

In order to migrate against flow, endothelial cells are first polarized against the direction of flow (Franco et al., 2015). Labeling the Golgi and nucleus allows endothelial cell polarization to be visualized. By comparing polarization to predicted flow patterns, endothelial cells were shown to be polarized against flow in the developing retina (Franco et al., 2015). A key player in the maintenance of the endothelial polarization, first identified in the retina, is the primary cilium of endothelial cells (Vion et al., 2018). Primary cilia are present on endothelial cells when exposed to low shear stress, whereas higher levels of shear stress often cause disassembly of the cilia (Iomini et al., 2004; Egorova et al., 2011; Ten Dijke et al., 2012). By a genetic deletion of the essential cilia component intraflagellar transport protein 88 (IFT88), Vion et al. (2018) described a random and premature regression of blood vessels related to an increase in migration and a reduced polarization of the endothelial cells.

**Figure 2:**
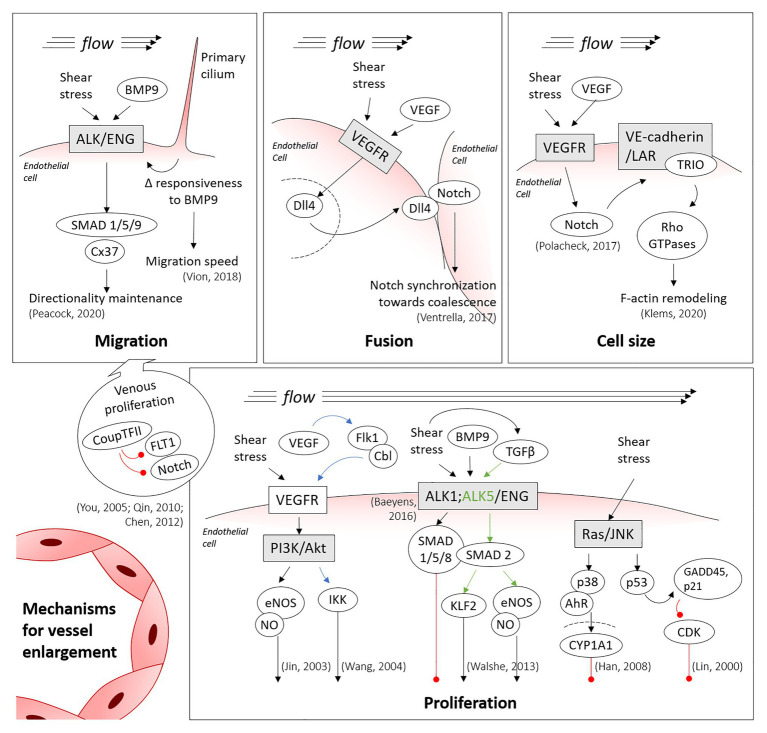
Main mechanisms involved in vessel enlargement. Summary of the shear-inducible mechanotransduction pathways involved in migration, fusion, hypertrophy and proliferation of endothelial cells, the processes directing vessel enlargement. Blue and green arrows are colored to clarify the trigger of specific downstream pathways, being shear stress involved in both cases. Red lines denote inhibition (thus, cell cycle arrest, in the proliferation panel). Discontinued lines denote the nuclei membranes.

The Bone Morphogenetic Protein/Activin receptor-like kinase 1/Endoglin (BMP/ALK1/ENG) pathway is also required for polarized migration in response to flow (Figure 2). Mutations in the *ENG* and *ACVRL1* genes (encoding for *ENG* and ALK1) cause Hereditary Hemorrhagic Telangiectasia (HHT), which is a genetic form of AVM development (McAllister et al., 1994; Johnson et al., 1996). Loss of directional migration has been reported for ENG (Jin et al., 2017) and ALK1 (Rochon et al., 2016) in mouse *ENG*-knockouts and in a zebrafish AVM model lacking *ALK1*, respectively. Recently, our group has identified that, under shear stress, SMAD1/5/9 (transcription factors in this pathway) control the expression of the gene (*GJA4*) for the gap junction protein Cx37 (Connexin37; Peacock et al., 2020). We also found that Cx37 has a critical role in the maintenance of the directionality of endothelial cell migration under flow. Interestingly, mechanotransduction by the primary cilium also functions through the ALK1 pathway, with the primary cilium increasing BMP9 responsiveness of endothelial cells under low shear stress, thereby decreasing their migration speed. This may prevent premature vascular regression under low shear stress processes, such as during the initial remodeling of the retinal vascular network (Vion et al., 2018).

Though regressing cells are a source of endothelial cells for enlarging vessels, the venous vascular bed also contributes cells. Notably, proliferation is higher in venous endothelial cells than in arterial endothelial cells (Red-Horse et al., 2010; Ehling et al., 2013) and, therefore, venous cells provide a source of cells for the migration model of vessel enlargement (Figure 2). Coup-TFII, which is one of the most important venous transcription factors, can repress the expression of Fms Related Receptor Tyrosine Kinase 1 (*FLT1*; also known as *VEGFR1*, Vascular Endothelial Growth Factor Receptor 1) and *Notch*, thereby promoting endothelial cell proliferation (You et al., 2005; Qin et al., 2010; Chen et al., 2012). In the mouse embryonic heart, for instance, coronary arteries have been shown to form from venous endothelial cells (Su et al., 2018). In the mouse retina, labeling tip cells permitted to observe the integration of the labeled cells in growing arteries, but rarely into the venous vascular bed (Xu et al., 2014; Pitulescu et al., 2017). This has led to the proposal that endothelial cells proliferate in veins, migrate from low shear stress veins through the capillary bed and then ultimately stop migrating in the arterial vascular bed because of high shear stress levels. They therefore accumulate in this region, inducing vessel enlargement (Red-Horse and Siekmann, 2019).

### Fusion

**Figure 3:**
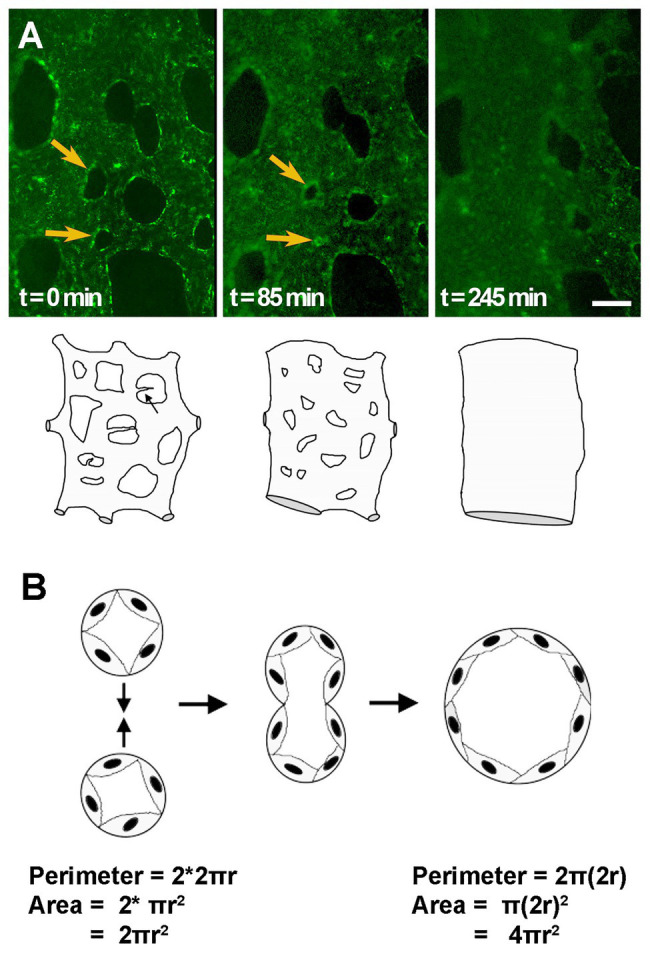
Vessel enlargement by fusion. **(A)** Capillary plexus vessels merge to form a larger vessel, as shown by time-lapse microscopy of AcLDL labeled vitelline vessels in quail embryos. Yellow arrows indicate avascular regions that become smaller and smaller until two adjacent vessels fuse. Cartoon exemplifies changes that can be observed by time-lapse microscopy. **(B)** Schematic of the fusion process in transverse view. The same number of cells, or perimeter length, leads to a doubling in the area for flow after fusion. Cartoons adapted from (Drake and Little, 1999).

Vessel fusion was first described over three decades ago but has received less attention than other mechanisms of vessel enlargement. Drake and Little (1995) were first to describe this process. By time-lapsing dorsal aorta development in avian embryos, they showed that a capillary bed initially formed along the length of the embryo proper and that with the onset of blood flow, these capillaries merge together forming larger and larger vessels (Figure 3A; Rupp et al., 2004). These observations were further confirmed with the development of transgenic quail embryos that allowed clear visualizations of the forming dorsal aorta [see movies S3 and S6, (Sato et al., 2010)]. Moreover, both the vitelline artery and vitelline vein were also reported to form by fusion of smaller capillaries in these transgenic quails (Sato et al., 2010).

Though the initial reports on fusion mainly focused on the dorsal aorta in avian embryos, this process was later shown to also occur in other models and other vascular beds. In mouse, time-lapse microscopy showed that fusion is the main mechanism by which yolk sac vessel enlarge, leading to periodic jumps in vessel diameter rather than smooth linear increases in vessel diameter (Udan et al., 2013). Here, fusion processes could be identified in the formation of both the vitelline artery and the vitelline vein in mouse. Quantification of sprouting/regression/fusion/splitting events during remodeling of the yolk sac vasculature shows that fusion is more common than vessel regression in the embryo, but slightly less common than angiogenesis (Chouinard-Pelletier et al., 2013). The overall number of fusion events is on the same order of magnitude as sprouting and regression events. The coalesce of a capillary bed along the embryonic midline has never been reported for the mouse dorsal aorta formation. However, the Semaphorin 3E (*SEMA3E*)^−/−^ embryo, involved in repulsive endothelial cell guidance, shows a transient phenotype whereby a plexus is present that eventually coalesces to paired dorsal aortae. This suggests that vessel coalescence may be retarded in these mutants and, therefore, observable (Meadows et al., 2013). Vessel enlargement in other organs has also been reported to occur by fusion, or coalescence, of smaller vessels, such as the central pancreatic duct, which develops from smaller capillary vessels joining and fusing into one large diameter vessel (Azizoglu et al., 2016).

Fusion is a much faster process to enlarge a vessel than either migration or proliferation. Within hours, two small vessels can merge leading to a doubling in the radius of the vessel, but a 4-fold increase in the cross-sectional area for flow (Figure 3B). This may explain why reports of fusion have been limited to embryonic vascular beds, where remodeling must occur much more rapidly than in more mature systems. It is also possible, thus, that difficulties in detecting fusion prevent its identification in other vascular beds. Morphologically, as vessels fuse, the avascular region between the two vessels becomes smaller and smaller, eventually creating “pillars” of avascular tissue that are identical to the pillars present in intussusceptive angiogenesis. As such, static images cannot differentiate fusion from intussusceptive angiogenesis. Indeed, fusion may only have been identified in embryonic vasculatures because these are the vascular beds where time-lapse microscopy at the resolution of capillaries is possible.

**Figure 4:**
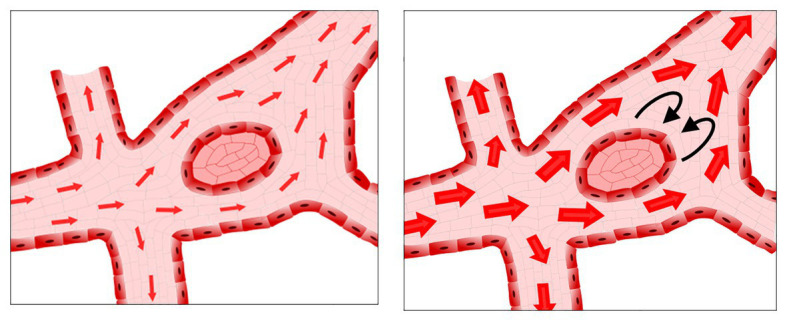
Possible low shear stress regions in the presence of increased flow rates. As flow increases, avascular regions can act as obstacles to the flow, resulting in regions of low shear stress and/or recirculation downstream from the avascular region.

Just as for migration, flow is essential for fusion to occur. In the aforementioned *SEMA3E*^−/−^ mutants, the timing of resolution of the unfused phenotype correlates to the onset of blood flow (Meadows et al., 2013). In normal vascular development, fusion occurs in regions with the highest flow, such as the region where the vitelline artery and vein are forming (Sato et al., 2010; Chouinard-Pelletier et al., 2013; Udan et al., 2013). Unexpectedly, if flow patterns are altered to reduce shear stress, an increased number of fusion events is observed (Chouinard-Pelletier et al., 2013). If shear stress levels are increased instead, the opposite occurs, and less fusion is present. Though these results may appear paradoxical, the increased flow in the region of the forming vitelline artery and vein do not necessarily mean that increased shear stress drives fusion. Shear stress relates not only to the flow velocity but also to geometry of the vessels. If an avascular region between two fusing vessels acts as an obstruction in the middle of a fast-flowing stream, then, as the velocity of that stream increases, shear stress will be increased on the upstream side of the avascular region but decreased on the downstream side (Figure 4). As such, gradients of shear stress may drive fusion events rather than just increases in the total amount or velocity of flow.

Given the difficulties in identifying fusion events, very little is known about the mechanism of fusion. There are various phenotypes, however, that present with a hyperfused vascular plexus. Inhibition of Notch in the embryonic yolk sac results in increased number of fusion events (Chouinard-Pelletier et al., 2013; Caolo et al., 2018). The increased amount of fusion observed during Notch inhibition can be rescued by increasing shear stress levels (Caolo et al., 2018). In the chick embryo yolk sac, exogenous vascular endothelial growth factor (VEGF) induced increased vascular fusion (Drake and Little, 1995). Sema3E signaling induces the expression of *sFLT1* (Zygmunt et al., 2011) and, as such, the *SEMA3E*^−/−^ should have reduced VEGF signaling. Though the interplay of VEGF and Notch signaling is well studied for sprouting angiogenesis (Eichmann and Simons, 2012), why they would induce fusion in other situations is a mystery. The key may lie in the role of VEGF in synchronizing Notch signaling (Figure 2). In somitogenesis, Notch is involved in synchronizing cells such that all cells cycle together and express the same genes together (Jiang et al., 2000). In endothelial cells, levels of Notch targeting genes also oscillate, but in an unsynchronized manner. Under higher levels of VEGF, however, Notch-induced gene expression synchronizes endothelial cells, favoring vessel enlargement rather than extension (Ubezio et al., 2016). The act of favoring “self” (staying together) rather than “other” (extending a sprout) has previously been proposed for the role of Notch target genes in boundary formation (Ventrella et al., 2017). In this model, Notch synchronization would create a situation where vessel coalesce with each other (i.e., fuse), as they prefer adhering to one another rather than remaining separate.

### Proliferation

Though no significant proliferation is observed during vessel enlargement in developmental models, whether the retina or the embryo, this is not the case for vessel enlargement in adult vascular beds. Chronic changes in flow in the adult lead to expansion of collateral blood vessels that restore normal blood flow levels. This process was termed arteriogenesis (Scholz et al., 2001). After partial coronary ligation model, endothelial cell proliferation is observed in enlarging vessels, peaking 3 weeks after implantation of the constrictor (Schaper et al., 1971). In hind limb ischemia models, significant endothelial cell and smooth muscle cell proliferation is observed in the enlarging vessels within 2–3 days of ligation (Scholz et al., 2000). Vessel enlargement by proliferation occurs over a period of days and weeks, not hours. Though endothelial cells are clearly proliferating within the growing arterioles (Scholz et al., 2000), this does not exclude a role for migration in collateral enlargement.

Vascular endothelial growth factor is most well known as a mitogen for endothelial cells proliferation. VEGF acts by binding to VEGF receptors, and this phosphorylates protein kinases that activate downstream Phosphoinositide 3-kinase (PI3K) and Mitogen-activated protein kinase (MAPK) pathways promoting proliferation (Figure 2). VEGF expression is triggered by hypoxia. The proliferation process involved in arteriole enlargement, however, does not respond to hypoxia (Deindl et al., 2001). In fact, although experimentally inhibited VEGF in femoral artery ligation (an occlusion model) results in inhibited arteriogenesis (Jacobi et al., 2004; Lloyd et al., 2005; Toyota et al., 2005), *VEGF* is not expressed in the tissue near the growing collaterals (Lee et al., 2004). Thus, although endothelial cells clearly proliferate within the growing arterioles (Scholz et al., 2000), the role of VEGF is unlikely to be needed for this local proliferation. Instead, VEGF may be needed to induce proliferation distal from the site of arteriogenesis, though it is also possible that VEGF could be responsible for the release of blood-marrow derived cells.

In the case of flow-induced remodeling, VEGF itself is also not a necessary ligand to activate PI3K/MAPK signaling pathways. Laminar flow induces a transphosphorylation of VEGF receptor 2 (VEGFR2) that activates the downstream PI3K pathway in a ligand-independent manner (Jin et al., 2003). This leads to the phosphorylation of Akt that has pleiotropic effects on proliferation. Akt phosphorylation by flow is highest at physiological shear stress levels between 10 and 20 dyn/cm^2^ (Dimmeler et al., 1998; Li et al., 2009), however, these levels of shear stress are known to induce quiescence and not proliferation in endothelial cells. VEGFR2 and Akt phosphorylation are, however, short-lived events that occur within the first 1–2 h of a change in shear stress (Shay-Salit et al., 2002; Guo et al., 2007). Oscillatory flow, which in contrast to unidirectional flow does induce increased proliferation, leads to prolonged VEGFR2 and Akt phosphorylation (Guo et al., 2007). Furthermore, unidirectional flow also activates other factors such as AMP-activated protein kinase (AMPK), which counteract the pro-proliferative signals (Guo et al., 2007).

Shear stress can also modulate endothelial cell proliferation through other signaling pathways. The phosphorylation of endothelial Nitric Oxide Synthase (eNOS) and its increased activity induced by shear stress was one of the first studied effects of mechanotransduction (Buga et al., 1991; Kuchan and Frangos, 1994). Nitric oxide (NO) not only induces vasodilation but also induces proliferation. In vasodilation, NO is produced by endothelial cells, diffuses to smooth muscle cells where it activates soluble guanylyl cyclase by binding to the heme group (Zhao et al., 1999). Estimates vary concerning the concentration at which this occurs, however, most publications have placed this between 5 and 100 nM (Chen et al., 2008). As a pro-proliferative compound, NO acts by controlling protein activation by reacting with cysteine residues to induce *S*-nitrosylation. NO leads to *S*-nitrosylation of MAPK phosphatase 7 (MKP7), rendering it inactive which then prevents the inactivation of c-Jun N-terminal Kinase 3 (JNK3; Pi et al., 2009). The concentrations at which NO induce proliferation are extremely low, in the pico to nanomolar ranges that occur due to release of NO by macrophages and endothelial cells (Ridnour et al., 2005; Thomas et al., 2008; Figure 2). At concentrations in the micromolar range, NO inhibits proliferation and induces cell cycle arrest in several cell types (Gooch et al., 1997; Heller et al., 1999).

Though the ALK1/ENG complex affects endothelial cell migration, this signaling pathway also has a key role in regulating the proliferation of endothelial cells (Goumans et al., 2002; David et al., 2007). The ALK1/ENG complex is, thus, a key component through which shear stress can block endothelial cells from entering the cell cycle (Baeyens et al., 2016; Figure 2). By recognizing both BMP9 and flow, the ALK1/ENG complex allows the modulation of vascular morphogenesis in response to flow (Baeyens et al., 2016). Mutations in *SMAD4* and in the Growth differentiation factor 2 (*GDF2*) genes (encoding for SMAD4 and BMP9) had been also reported later on to cause variants of HHT. Both the endothelial-specific ablation of SMAD4 (a transcription factor in the ALK1/ENG pathway) and of ENG show increased proliferation of endothelial cells within the developing shunt (Ola et al., 2018; Tual-Chalot et al., 2020). The later, however, has been described to involve the VEGF signaling pathway (Tual-Chalot et al., 2020).

Transforming Growth Factor-β (TGF-β) is involved in the maintenance of the endothelium in a nonactivated state (Walshe et al., 2009) and protecting it from aberrant permeability and perfusion, and from apoptosis (Walshe et al., 2011). Interestingly, experiments on HUVECs demonstrated that shear stress activates TGF-β, leading to downstream activation of Krüppel-Like Factor 2 (KLF2) and eNOS in an ALK5 dependent manner (Walshe et al., 2013; Figure 2). Moreover, TGF-β malfunction through the SMAD signaling pathway has been linked to diverse cerebrovascular diseases related to aberrant endothelial cell proliferation (including HHT), as reviewed by Zhang and Yang (2020).

Flow can also modulate the endothelial cell cycle through other pathways. For example, in bovine aortic endothelial cells, 24 h of laminar shear stress (3–12 dyn/cm^2^) activated the phosphorylation of p53 protein through the JNK pathway. The increased levels of p53 in turn activated Growth Arrest and DNA Damage 45 (GADD45) and p21 proteins, inhibiting the Cyclin-Dependent Kinase (CDK) and, thus, arresting endothelial cell proliferation (Lin et al., 2000; Figure 2). Transcription factor Aryl hydrocarbon receptor (AhR) is also sensitive to shear stress. In this case, laminar shear stress between 6 and 15 dyn/cm^2^ induced –likely also through the JNK/p38 pathway– the expression and the translocation of AhR into the nucleus. In the nucleus, AhR promotes an increase in Cytochrome P450 Family 1 Subfamily A Member 1 (CYP1A1) expression and the subsequent shear stress-induced arrest of the cell cycle (Han et al., 2008; Figure 2).

### Hypertrophy

Endothelial cells have an amazing ability to change their cell size. Vessel enlargement by hypertrophy results in an increase in the size of individual endothelial cells and can extremely rapidly increase vessel diameter. Endothelial cell density in vessels is very high, meaning that there is a large potential for growth purely by altering their size.

In normal embryonic development, no differences in cell density are observed along vessel growth based on somite stage. Nonetheless, between large and small vessels a 30% reduction in endothelial cell density was reported (Udan et al., 2013). The difference, however, was not large enough to explain the difference in vessel diameter, suggesting that size of endothelial cells might be involved. Similarly, in the retina there is no overall change in endothelial cells density as large vessels form, but in this case, the endothelial cell density was not compared between large and small vessels (Franco et al., 2015). In transgenic zebrafish embryos with constitutive or inducible expression of Placental Growth Factor (*PLGF*) under control of a somite muscle-specific promoter (PLGF*^musc^*), cell size was found to contribute to vessel enlargement but, importantly, alone could not account for the diameter increase (Klems et al., 2020). As such, in normal vascular development as well, endothelial cell hypertrophy appears to contribute to vessel enlargement but never acts alone.

Though endothelial cell size increases appear to play a lesser role in developmental vessel enlargement, this process could still contribute to pathological vessel enlargement. In an AVM model of constitutive expression of active *Notch4*, pathological vessel enlargement occurs not due to an increase in endothelial cell density nor proliferation but, instead, due to an increased size of individual endothelial cells (Murphy et al., 2014). In zebrafish embryos, TRIO (Trio Rho Guanine Nucleotide Exchange Factor) activation, which in turn activates Ras homologous (Rho) GTPases, also leads to increased cell size causing an enlargement in arterial caliber (Klems et al., 2020). Interestingly, these results may be linked because shear-induced Notch activation has been shown to regulate and activate the assembly of a VE-Cadherin/LAR (leukocyte antigen-related protein tyrosine phosphatase)/TRIO complex (Polacheck et al., 2017; Figure 2). Other AVM models, such as the endothelial cell specific knockout of ENG (Choi et al., 2014), also present with an increase in endothelial cell size, both in zebrafish and mouse embryos (Sugden et al., 2017). Overall, however, the contribution of cell size changes to vessel growth is rarely assessed.

## Enlargement of Veins

The process of vessel enlargement is referred to as arteriogenesis when it occurs in the mature arterial vascular network, but veins can also increase in diameter. The process of increasing venous diameter is so understudied that it lacks a name, though it is occasionally referred to as venogenesis.

In vascular development, fusion has been reported as the predominant mechanism of venous vessel enlargement in both mouse and chicken embryos (le Noble et al., 2004; Udan et al., 2013). In the chick embryo, the vitelline vein arises from a region that is genetically arterial before vessel enlargement begins (le Noble et al., 2004). In the developing retina, endothelial specific ablation of Cell Division Control protein 42 (*CDC42* homolog) impairs migration, and enlarged veins and capillaries form without arterial enlargement (Lavina et al., 2018). This increased diameter was attributed to the presence of an increase in the number of endothelial cells per vessel length, without an increase in venous proliferation.

Understanding the process of vein enlargement requires mutants that specifically show changes in the diameter of veins. As such, somatic mutations that lead to venous malformations can be especially informative. Venous malformations are enlarged veins that present with few mural cells. The most well studied somatic mutations in venous malformation are the ones related to the Tyrosine-protein kinase (Tie2) receptor (Limaye et al., 2009; Soblet et al., 2013). Constitutive ablation of *TIE2* leads to embryonic lethality at E10.5 (Sato et al., 1995). If *TIE2* is ablated at later stages, however, arteries continue to form but veins do not (Chu et al., 2016). This is associated with a loss of venous markers [EphB4 (Ephrin type-B receptor 4), APJ (apelin receptor)] without any increase in arterial markers, indicating that this may be a defect in venous specification rather than vessel enlargement. Constitutively active forms of Tie2 that replicate somatic *TIE2* mutations found in venous malformations, cause increased migration but loss of proper polarization in endothelial cells *in vitro* (Cai et al., 2019). However, it is not clear whether loss of proper cell identity or improper migration lead to vessel enlargement in venous malformations.

Though venous malformations arise from veins, some argue that all AVMs originate from venous endothelial cells. In biopsies from patients with telangiectasias, the enlargement of post-capillary venules precedes AVM formation (Braverman et al., 1990). In mouse models, exogenous expression of activated Notch4 induces AVM formation (Murphy et al., 2014). However, when this expression is limited to arteries, no AVMs form (Murphy et al., 2014). Deletion of the ENG only in venous and capillary endothelial cells results in the same rate of AVM formation as for deletion in all endothelial cells (Singh et al., 2020). Conversely, in retinas of an endothelial-specific *ENG* knockout model, imaging of developing AVMs showed that these initiated in arterioles and grew toward the venous vasculature (Jin et al., 2017). Although this would suggest an arterialization of the capillaries, in mouse models of AVM formation, the AVMs themselves express venous markers and downregulate arterial marker (Ola et al., 2018).

Another venous malformation that leads to increased venogenesis is varicose vein development. Though most research on vessel enlargement focuses on the role of shear stress as the stimuli for enlargement, varicose vein development occurs due to defective valves leading to an increase in hydrodynamic pressure (Welkie et al., 1992; Nicolaides et al., 1993). It should be noted, however, that the increased pressure in veins also leads to altered shear stress. On a cellular level, varicose vein development involves activation of endothelial cells leading to immune cell recruitment, increased vessel leakiness and loss of smooth muscle cells (Segiet et al., 2015). Varicose veins have upregulated Notch pathway genes like Delta Like Canonical Notch Ligand 4 (*DLL4*), Hairy/enhancer-of-split related with YRPW motif protein 2 (*HEY2*), and *EPHB2* (Surendran et al., 2016). Smooth muscle cells become enlarged and surrounded by an increased amount of extracellular matrix, suggesting that they de-differentiate into a synthetic phenotype (Wali and Eid, 2001). Matrix Metalloproteinases (MMPs) and Tissue Inhibitors of Metalloproteinases (TIMPs) play an important role in the development of varicose veins. Increases in TIMP-1 levels and in the TIMP/MMP-2 ratio lead to an increase in extracellular matrix deposition and a decrease in degradation processes (Badier-Commander et al., 2000). The mechanism of enlargement for varicose vein is, therefore, much more akin to collateral vessel enlargement than to vessel enlargement in developmental models.

## Differences Between Vessel Enlargement in the Adult and Embryo

Vessel enlargement occurs during embryonic development but continues to occur in the adult vascular networks. Any time the vasculature is exposed to a chronic change in flow, the vasculature adapts through either enlargement or inward remodeling to accommodate the altered flow. The most common experimental model for vessel enlargement in the adult is the remodeling of collateral vessels after occlusion, whether by partial occlusion of a coronary artery or by ligation of the femoral or middle cerebral artery. Other models, such as arteriovenous fistulas, have also been used to study the mechanisms of vessel enlargement. Vessel enlargement post-natally is inherently different than in developmental models, such as the retina, since post-natal enlargement requires the degradation of an existing basement membrane as well as the detachment and proliferation of mural cells. Furthermore, the remodeling of adult vessels, such as the collaterals, is initiated by endothelial cell activation, leukocyte invasion, and proliferation of vascular cells (Ma and Bai, 2020). This leads to the question, what parallels exist between vessel enlargement in developmental models and in adult models? And though there may be more than one way in which a vessel enlarges, it is likely that there will be common components from which we can gain significant insight.

### Endothelial Cell Activation

In femoral artery ligation, arteriogenesis occurs far away from where ischemia is occurring (Resnick et al., 2003; Pipp et al., 2004). As such, just as with developmental angiogenesis, it is a process driven by shear stress. Just after coronary occlusion, the endothelial cells in the collateral vessel lose alignment with flow and take on a bulged appearance (Cai and Schaper, 2008). These cells increase DNA synthesis (Schaper et al., 1971; Pasyk et al., 1982) and proliferation, as indicated by Bromodeoxyuridine (BrdU) incorporation or Ki67 staining (Arras et al., 1998b; Wolf et al., 1998). Interestingly, these are all behaviors associated with low or recirculating shear stress patterns and not with increased shear stress levels. Adult endothelial cells, however, are adapted to the flow to which they are exposed and become activated in response to altered flow (Ward et al., 2020). Furthermore, though physiological shear stress reduces activation and proliferation, when shear levels are extremely elevated (above 30 dyn/cm^2^ in humans), the response is outward remodeling (Dolan et al., 2013). Thus, difference between developmental and adult remodeling may arise from either one of the following facts. On the one hand, developmental vasculature is naïve and therefore, it is not adapting to its “expected” flow; on the other hand, the stimulus for remodeling is a physiological level of shear stress (i.e., 15 dyn/cm^2^) for vessel enlargement during development, but an acute non-physiological level of shear stress (above 30 dyn/cm^2^) in models of vessel enlargement in the adult.

The activated endothelium produces NO that is essential for collateral growth. Both eNOS and iNOS (respectively, endothelial and inducible NOS) are upregulated in remodeling collateral vessel (Cai et al., 2004; Yang et al., 2015). When NO production is inhibited with L-NAME [N(G)-nitro L-arginine methyl ester], there is an almost complete inhibition of collateral enlargement (Eitenmuller et al., 2006; Park et al., 2010). It is not clear, however, whether this is due to a true inhibition of growth or related to increased vasoconstriction (Cai and Schaper, 2008). Endothelial NOS itself is involved in maintaining collateral vessel under physiological conditions. Mice that lack eNOS are born with a normal number of collateral vessels in the brain, but the number of these vessels decreases over the first 6 months of life as compared to age-matched controls (Dai and Faber, 2010). The primary role of NO in vessel enlargement is in the recruitment of immune cells (Park et al., 2010). Delivery of NO donors induces VE-Cadherin disassembly that is necessary for immune cell recruitment. Conversely, NO inhibitor L-NAME prevents increased vessel permeability and immune cell recruitment after vessel occlusion (Yang et al., 2015). Though NO is critical in vessel enlargement in post-natal stages, it has not been extensively investigated during development. The triple knockout of NOS enzymes is born at normal mendelian frequency with no reported vascular defects at birth (Morishita et al., 2005). Conversely, however, culture of E8.5 mouse embryos with the NO inhibitor L-NMMA [N(G)-monomethyl L-arginine] prevents the formation of large vessels in the yolk sac vasculature (Nath et al., 2004). NO influences endothelial cell proliferation (Morbidelli et al., 1996) and migration (Noiri et al., 1998). As such, NO could have a role in several of the mechanisms by which vessel enlarge.

### Immune Cells and Matrix Degradation

Another difference between adult and developmental vessel enlargement is the involvement of immune cells. Immune cells, especially monocytes and macrophages, are essential for adult collateral growth and vessel enlargement in general (Arras et al., 1998a; Voskuil et al., 2003). Inhibiting monocyte recruitment in the adult impairs arteriogenesis during collateral remodeling (Heil et al., 2002, 2004). Recruited monocytes produce Tumor necrosis factor-α (TNF-α) and VEGF, which induces endothelial and smooth muscle cell mitoses (Schaper and Ito, 1996).

Though essential when vessel enlarge post-natally, functional immune cells may not be as present in vessel enlargement occurring just after the onset of flow in the embryo. The first immune cells form at E8.5 in the form of erythromyeloid progenitor and primitive macrophages (Gomez Perdiguero et al., 2015), and no functional role for these progenitors has been established until much later in development. At Hamburger Hamilton stage 18 in avian embryos (equivalent to E12.5–13.5 in mouse), circulating phagocytic cells are recruited to sites of vascular remodeling (Al-Roubaie et al., 2012). In the zebrafish embryo, depletion of myeloid cells using a *pu.1* morpholino inhibits collateral growth in the *gridlock* mutant embryo (Gray et al., 2007). However, both these reports were for embryos at stages much older than the ones that gave the results showing vessel enlargement by migration and/or fusion (Chouinard-Pelletier et al., 2013). The retinal vasculature does form post-natally, when resident myeloid cells are present in the retina (Haupt et al., 2019). Ablation of macrophages using chlodronate liposomes results in a dramatic loss of vascular density, which makes it difficult to assess whether vessel enlargement itself is affected (Checchin et al., 2006).

One of the roles of immune cells in arteriogenesis is to degrade the basement membrane. During arteriogenesis, the elastic lamina is broken down by MMPs to give the vessels room to expand (Haas et al., 2007; Dodd et al., 2011). Inflammatory cells are also an important source of MMPs, as well as of other proteases. Macrophages secrete cytokines that induce MMP expression by endothelial cells (Galis et al., 1994a). Vessel enlargement in response to arteriovenous fistula induces a more than 1700-fold increase in MMP-2, along with a 12–60-fold increase in MMP-9, Membrane-type 1 MMP (MT1-MMP), and TIMP-2 (Sho et al., 2002). These increases correlate to the timing of elastic lamina degradation (Sho et al., 2002). Notably, MMPs are produced as inactive zymogens and, in addition, TIMPs can inhibit their activity. Therefore, increased expression of MMPs does not necessarily indicate increased activity. Indeed, many MMPs are constitutively expressed by endothelial cells and smooth muscle cells (Hanemaaijer et al., 1993; Galis et al., 1994a) but show no enzymatic activity until activated by disease (Galis et al., 1994b, 1995). Though essential in adult remodeling, no single mutant of MMPs has shown defects during embryogenesis. The double mutant of MMP2 and MT1-MMP does die perinatally, with a defect in the formation of vessels with wider diameters (Oh et al., 2004). This is, however, a very late stage of vascular development, after initial vascular remodeling has occurred.

One of the effects of immune cell recruitment and matrix degradation is an increase in permeability. Middle cerebral artery occlusion leads to an increase in permeability in the blood-brain-barrier to large molecules such as fibrinogen, Immunoglobulin G (IgG) or nanoparticles within 2–4 h of the occlusion (Okada et al., 1994; Fischer et al., 2002). Degradation is necessary for this increase in permeability, as inhibiting MMPs with BB-1101 prevents permeability increases immediately after middle cerebral artery occlusion (Rosenberg et al., 1998).

The presence of this extensive basement membrane is one of the main reasons that post-natal vessel enlargement is unlike to occur by vascular fusion. The presence of extensive matrix and mural cells from the arterioles onward would be a physical barrier for fusion. Hence, vascular fusion could only occur on the capillary level allowing arterioles to grow, which would then have to further increase in diameter by combination with another mechanism.

## Conclusion

Vessel enlargement plays a critical role both during development as well as in the adult vasculature, with a high capacity for adapting to flow changes. Through an extremely fast responsiveness and interconnected processes such as fusion, endothelial cell migration and proliferation, vessels reshape in order to accommodate changes in flow rates such as to restore physiological levels. Because these processes are so much present along all the vasculature lifetime, dysregulations may entail critical pathologies. However, more and more pathways and molecular interconnections are being uncovered, shedding light to a better understanding and control over these pathologies.

## Author Contributions

LG-R and EAVJ contributed to writing, editing, and making figures. All authors contributed to the article and approved the submitted version.

### Conflict of Interest

The authors declare that the research was conducted in the absence of any commercial or financial relationships that could be construed as a potential conflict of interest.
